# On Topological Structures of Fuzzy Parametrized Soft Sets

**DOI:** 10.1155/2014/164176

**Published:** 2014-04-28

**Authors:** Serkan Atmaca, İdris Zorlutuna

**Affiliations:** Department of Mathematics, Cumhuriyet University, 58140 Sivas, Turkey

## Abstract

We introduce the topological structure of fuzzy parametrized soft sets and fuzzy parametrized soft mappings. We define the notion of quasi-coincidence for fuzzy parametrized soft sets and investigated its basic properties. We study the closure, interior, base, continuity, and compactness and properties of these concepts in fuzzy parametrized soft topological spaces.

## 1. Introduction

In 1965, after Zadeh [1] generalized the usual notion of a set with the introduction of fuzzy set, the fuzzy set was carried out in the areas of general theories and applied to many real life problems in uncertain, ambiguous environment. In this manner, in 1968, Chang [2] gave the definition of fuzzy topology and introduced the many topological notions in fuzzy setting.

In 1999, Molodtsov [3] introduced the concept of soft set theory which is a completely new approach for modelling uncertainty and pointed out several directions for the applications of soft sets, such as game theory, perron integrations, and smoothness of functions. To improve this concept, many researchers applied this concept on topological spaces (e.g., [4–9]), group theory, ring theory (e.g., [10–14]), and also decision making problems (e.g., [15–18]).

Recently, researchers have combined fuzzy set and soft set to generalize the spaces and to solve more complicated problems. By this way, many interesting applications of soft set theory have been expanded. First combination of fuzzy set and soft set is fuzzy soft set and it was given by Maji et al. [19]. Then fuzzy soft set theory has been applied in several directions, such as topology (e.g., [20–23]), various algebraic structures (e.g., [24, 25]), and especially decision making (e.g., [26–29]). Another combination of fuzzy set and soft set was given by Ça$\overset{˘}{\text{g}}$man et al. [30] who called it fuzzy parametrized soft set (for short FP-soft set). In that paper, Ça$\overset{˘}{\text{g}}$man et al. defined operations on FP-soft sets and improved several results. After that, Ça$\overset{˘}{\text{g}}$man and Deli [31, 32] applied FP-soft sets to define some decision making methods and applied these methods to problems that contain uncertainties and fuzzy object.

In the present paper, we consider the topological structure of FP-soft sets. Firstly, we give some basic ideas of FP-soft sets and also studied results. We define FP-soft quasi-coincidence, as a generalization of quasi-coincidence in fuzzy manner [33], and use this notion to characterize concepts of FP-soft closure and FP-soft base in FP-soft topological spaces. We also introduce the notion of mapping on FP-soft classes and investigate the properties of FP-soft images and FP-soft inverse images of FP-soft sets. We define FP-soft topology in Chang's sense. We study the FP-soft closure and FP-soft interior operators and properties of these concepts. Lastly we define FP-soft continuous mappings and we show that image of a FP-soft compact space is also FP-soft compact.

## 2. Preliminaries

Throughout this paper *X* denotes initial universe, *E* denotes the set of all possible parameters which are attributes, characteristic, or properties of the objects in *X*, and the set of all subsets of *X* will be denoted by *P*(*X*).

**Definition 1 (see [1])**

A fuzzy set *A* in *X* is a function defined as follows:

(1) $$\begin{array}{c} A=\left\{\frac{\mu_{A}\left(x\right)}{x}:x\in X\right\}, \end{array}$$

where *μ*_*A*_ : *X* → [0,1].

Here *μ*_*A*_ is called the membership function of *A*, and the value *μ*_*A*_(*x*) is called the grade of membership of *x* ∈ *X*. This value represents the degree of *x* belonging to the fuzzy set *A*.

A fuzzy point in *X*, whose value is *α*  (0 < *α* ≤ 1) at the support *x* ∈ *X*, is denoted by *x*_*α*_. A fuzzy point *x*_*α*_ ∈ *A*, where *A* is fuzzy set in *X* if *α* ≤ *μ*_*A*_(*x*).

**Definition 2 (see [3])**

A pair (*F*, *E*) is called a soft set over *X* if *F* is a mapping defined by *F* : *E* → *P*(*X*).

In other words, a soft set is a parametrized family of subsets of the set *X*. Each set *F*(*e*), *e* ∈ *E*, from this family may be considered as the set of *e*-elements of the soft set (*F*, *E*).

**Definition 3 (see [30])**

Let *A* be a fuzzy set over *E*. A FP-soft set *F*_*A*_ on the universe *X* is defined as follows:

(2) FA={(μA(e)e,fA(e)):e∈E,fA(e)∈P(X),μA(e)∈[0,1]},

where the function *f*_*A*_ : *E* → *P*(*X*) is called approximate function such that *f*_*A*_(*e*) = *⌀* if *μ*_*A*_(*e*) = 0, and the function *μ*_*A*_ : *E* → [0,1] is called membership function of the set *A*.

From now on, the set of all FP-soft sets over *X* will be denoted by FPS(*X*, *E*).

**Definition 4 (see [30])**

Let *F*_*A*_ ∈ FPS(*X*, *E*).

1. *F*_*A*_ is called the empty FP-soft set if *μ*_*A*_(*e*) = 0 for every *e* ∈ *E*, denoted by *F*_*⌀*_.
2. *F*_*A*_ is called *A*-universal FP-soft set if *μ*_*A*_(*e*) = 1 and *f*_*A*_(*e*) = *X* for all *e* ∈ *A*, denoted by $F_{\tilde{A}}$.
3. If *A* = *E*, then *A*-universal FP-soft set is called universal FP-soft set, denoted by $F_{\tilde{E}}$.

**Definition 5 (see [30])**

Let *F*_*A*_, *F*_*B*_ ∈ FPS(*X*, *E*).

1. *F*_*A*_ is called a FP-soft subset of *F*_*B*_ if *A* ≤ *B* and *f*_*A*_(*e*)⊆*f*_*B*_(*e*) for every *e* ∈ *E* and one writes $F_{A}\tilde{\subset }\;F_{B}$.
2. *F*_*A*_ and *F*_*B*_ are said to be equal, denoted by *F*_*A*_ = *F*_*B*_ if $F_{A}\tilde{\subset }F_{B}$ and $F_{B}\tilde{\subset }\;F_{A}$.
3. The union of *F*_*A*_ and *F*_*B*_, denoted by $F_{A}\tilde{\cup }F_{B}$, is the FP-soft set, defined by the membership and approximate functions *μ*_*A*∪*B*_(*e*) = max⁡{*μ*_*A*_(*e*), *μ*_*B*_(*e*)} and *f*_*A*∪*B*_(*e*) = *f*_*A*_(*e*) ∪ *g*_*B*_(*e*) for every *e* ∈ *E*, respectively.
4. The intersection of *F*_*A*_ and *F*_*B*_, denoted by $F_{A}\;\tilde{\cap }\;G_{B}$, is the FP-soft set, defined by the membership and approximate functions *μ*_*A*∩*B*_(*e*) = min⁡{*μ*_*A*_(*e*), *μ*_*B*_(*e*)} and *f*_*A*∩*B*_(*e*) = *f*_*A*_(*e*)∩*g*_*B*_(*e*) for every *e* ∈ *E*, respectively.

**Definition 6 (see [30])**

Let *F*_*A*_ ∈ FPS(*X*, *E*). Then the complement of *F*_*A*_, denoted by *F*_*A*_^*c*^, is the FP-soft set, defined by the membership and approximate functions *μ*_*A*^*c*^_(*e*) = 1 − *μ*_*A*_(*e*) and *f*_*A*_^*c*^(*e*) = *X* − *f*_*A*_(*e*) for every *e* ∈ *E*, respectively.

Clearly (*F*_*A*_^*c*^)^*c*^ = *F*_*A*_, $\;F_{\tilde{E}}^{c}=F_{⌀}$, and $F_{⌀}^{c}=F_{\tilde{E}}$.

**Proposition 7 (see [30])**

Let *F*_*A*_, *F*_*B*_, and *F*_*C*_ ∈ *FPS*(*X*, *E*). Then

1. ${\left(F_{A}\;\tilde{\cup }\;F_{B}\right)}^{c}=F_{A}^{c}\;\tilde{\cap }\;F_{B}^{c}$ *;*
2. ${\left(F_{A}\;\tilde{\cap }\;F_{B}\right)}^{c}=F_{A}^{c}\;\tilde{\cup }\;F_{B}^{c}$ *;*
3. $F_{A}\;\tilde{\cap }\;F_{A}=F_{A}$, $F_{A}\;\tilde{\cup }\;F_{A}=F_{A}$ *;*
4. $F_{A}\;\tilde{\cap }\;F_{⌀}=F_{⌀}$, $F_{A}\;\tilde{\cap }\;F_{\tilde{E}}=F_{A}$ *;*
5. $F_{A}\;\tilde{\cap }\;F_{B}=F_{B}\;\tilde{\cap }\;F_{A}$, $F_{A}\;\tilde{\cup }\;F_{B}=F_{B}\;\tilde{\cup }\;F_{A}$ *;*
6. $F_{A}\tilde{\cap }(F_{B}\;\tilde{\cap }\;F_{C})=$ $(F_{A}\;\tilde{\cap }\;F_{B})\tilde{\cap }F_{C}$, $F_{A}\tilde{\cup }(F_{B}\;\tilde{\cup }\;F_{C})=$ $(F_{A}\;\tilde{\cup }\;F_{B})\tilde{\cup }F_{C}$ *;*
7. $F_{A}\tilde{\cup }F_{⌀}=F_{A}$, $F_{A}\tilde{\cup }F_{\tilde{E}}=F_{\tilde{E}}$.

## 3. Some Properties of FP-Soft Sets and FP-Soft Mappings

**Definition 8**

Let *J* be an arbitrary index set and *F*_*A*_*i*__ ∈ *FPS*(*X*, *E*) for all *i* ∈ *J*.

1. The union of *F*_*A*_*i*__'s, denoted by ${\tilde{\cup }}_{i\in J}^{}F_{A_{i}}$, is the FP-soft set, defined by the membership and approximate functions *μ*_∪_*i*∈*J*_*A*_*i*__(*e*) = sup⁡_*i*∈*J*_⁡{*μ*_*A*_*i*__(*e*)} and *f*_∪_*i*∈*J*_*A*_*i*__(*e*) = ∪_*i*∈*J*_ *f*_*A*_*i*__(*e*) for every *e* ∈ *E*, respectively.
2. The intersection of *F*_*A*_*i*__'s, denoted by ${\tilde{\cap }}_{i\in J}^{}F_{A_{i}}$, is the FP-soft set, defined by the membership and approximate functions *μ*_∩_*i*∈*J*_*A*_*i*__(*e*) = inf⁡_*i*∈*J*_⁡{*μ*_*A*_*i*__(*e*)} and *f*_∩_*i*∈*J*_*A*_*i*__(*e*) = ∩_*i*∈*J*_ *f*_*A*_*i*__(*e*) for every *e* ∈ *E*, respectively.

**Proposition 9**

Let *J* be an arbitrary index set and *F*_*A*_*i*__ ∈ FPS(*X*, *E*) for all *i* ∈ *J*. Then

1. ${\left({\tilde{\cup }}_{i\in J}^{}F_{A_{i}}\right)}^{c}={\tilde{\cap }}_{i\in J}^{}F_{A_{i}}^{c}$ *;*
2. ${\left({\tilde{\cap }}_{i\in J}^{}F_{A_{i}}\right)}^{c}={\tilde{\cup }}_{i\in J}^{}F_{A_{i}}^{c}$.

**Proof**

(1) Put $F_{B}={\left({\tilde{\cup }}_{i\in J}^{}F_{A_{i}}\right)}^{c}$ and $F_{C}={\tilde{\cap }}_{i\in J}^{}F_{A_{i}}^{c}$. Then for all *e* ∈ *E*,

(3) μB(e)=1−μ∪i∈JAi(e)=1−sup⁡i∈J{μAi(e)}=inf⁡i∈J{1−μAi(e)}=inf⁡i∈J{μAic(e)}=μC(e),fB(e)=X−f∪i∈JAi(e)=X−∪i∈JfAi(e)=∩i∈J(X−fAi(e))=∩i∈JfAic(e)=f∩i∈JAic(e)=fC(e).

This completes the proof. The other can be proved similarly.

**Definition 10**

The FP-soft set *F*_*A*_ ∈ FPS(*X*, *E*) is called FP-soft point if *A* is fuzzy singleton and *f*_*A*_(*e*) ∈ *P*(*X*) for *e* ∈ supp⁡*A*. If *A* = {*e*}, *μ*_*A*_(*e*) = *α* ∈ (0,1], then one denotes this FP-soft point by *e*_*α*_^*f*^.

**Definition 11**

Let *e*_*α*_^*f*^, *F*_*A*_ ∈ FPS(*X*, *E*). One says that $e_{\alpha }^{f}\;\tilde{\in }\;F_{A}$ read as *e*_*α*_^*f*^ belongs to the FP-soft set *F*_*A*_ if *α* ≤ *μ*_*A*_(*e*) and *f*(*e*)⊆*f*_*A*_(*e*).

**Proposition 12**

Every nonempty FP-soft set *F*_*A*_ can be expressed as the union of all the FP-soft points which belong to *F*_*A*_.

**Proof**

This follows from the fact that any fuzzy set is the union of fuzzy points which belong to it [33].

**Definition 13**

Let *F*_*A*_,  *F*_*B*_ ∈ FPS(*X*, *E*). *F*_*A*_ is said to be FP-soft quasi-coincident with *F*_*B*_, denoted by *F*_*A*_ *qF*_*B*_, if there exists *e* ∈ *E* such that *μ*_*A*_(*e*) + *μ*_*B*_(*e*) > 1 or *f*_*A*_(*e*) is not subset of *f*_*B*_^*c*^(*e*). If *F*_*A*_ is not FP-soft quasi-coincident with *F*_*B*_, then one writes $F_{A}\bar{q}F_{B}$.

**Definition 14**

Let *e*_*α*_^*f*^,  *F*_*A*_ ∈ FPS(*X*, *E*). *e*_*α*_^*f*^ is said to be FP-soft quasi-coincident with *F*_*A*_, denoted by *e*_*α*_^*f*^ *qF*_*A*_, if *α* + *μ*_*A*_(*e*) > 1 or *f*(*e*) is not subset of *f*_*A*_^*c*^(*e*). If *e*_*α*_^*f*^ is not FP-soft quasi-coincident with *F*_*A*_, then one writes $e_{\alpha }^{f}\bar{q}F_{A}$.

**Proposition 15**

Let *F*_*A*_, *F*_*B*_ ∈ FPS(*X*, *E*). Then the following are true: 

1. $F_{A}\tilde{\subseteq }F_{B}\Leftrightarrow F_{A}\bar{q}F_{B}^{c}$ *;*
2. $F_{A}qF_{B}\Rightarrow F_{A}\tilde{\cap }F_{B}\neq F_{⌀}$.
3. $F_{A}\bar{q}F_{A}^{c}$ *;*
4. *F*_*A*_ *qF*_*B*_⇔* there exists an* $e_{\alpha }^{f}\tilde{\in }F_{A}$ *such that e*_*α*_^*f*^ *qF*_*B*_ *;*
5. $e_{\alpha }^{f}\tilde{\in }F_{A}^{c}\Leftrightarrow e_{\alpha }^{f}\bar{q}F_{A}$ *;*
6. $F_{A}\tilde{\subseteq }F_{B}\Rightarrow$ *if e*_*α*_^*f*^ *qF*_*A*_ *, then e*_*α*_^*f*^ *qF*_*B*_ *for all e*_*α*_^*f*^ ∈ *FPS*(*X*, *E*)*;*

**Proof**

(1) Consider

(4) FA⊆~FB⟺∀e∈E,  μA(e)≤μB(e), fA(e)⊆fB(e)⟺∀e∈E,  μA(e)−μB(e)≤0, fA(e)⊆fB(e)⟺∀e∈E,  μA(e)+1−μB(e)≤1, fA(e)⊆fB(e)⟺FAq¯FBc.

(2) Let *F*_*A*_ *qF*_*B*_. Then there exists an *e* ∈ *E* such that *μ*_*A*_(*e*) + *μ*_*B*_(*e*) > 1 or *f*_*A*_(*e*) is not subset of *f*_*B*_^*c*^(*e*). If *μ*_*A*_(*e*) + *μ*_*B*_(*e*) > 1, *A*∧*B* ≠ 0_*E*_ and the proof is easy. If *f*_*A*_(*e*) is not subset of *f*_*B*_^*c*^(*e*), then *f*_*A*_(*e*)∩*f*_*B*_(*e*) ≠ *⌀*. Hence $F_{A}\tilde{\cap }F_{B}\neq F_{⌀}$.

(3) Suppose *F*_*A*_ *qF*_*A*_^*c*^. Then there exists *e* ∈ *E* such that *μ*_*A*_(*e*) + *μ*_*A*^*c*^_(*e*) > 1 or *f*_*A*_(*e*) is not subset of (*f*_*A*_^*c*^(*e*))^*c*^. But this is impossible.

(4) If *F*_*A*_ *qF*_*B*_, then there exists an *e* ∈ *E* such that *μ*_*A*_(*e*) + *μ*_*B*_(*e*) > 1 or *f*_*A*_(*e*) is not subset of *f*_*B*_^*c*^(*e*). Put *α* = *μ*_*A*_(*e*) and *f*(*e*) = *f*_*A*_(*e*). Then we have $e_{\alpha }^{f}\tilde{\in }F_{A}$ and *e*_*α*_^*f*^ *qF*_*B*_.

Conversely, suppose *e*_*α*_^*f*^ *qF*_*B*_ for some $e_{\alpha }^{f}\tilde{\in }F_{A}$. Then *α* + *μ*_*B*_(*e*) > 1 or *f*(*e*) is not subset of *f*_*B*_^*c*^(*e*). Therefore, we have *μ*_*A*_(*e*) + *μ*_*B*_(*e*) > 1 or *f*_*A*_(*e*) is not subset of *f*_*B*_^*c*^(*e*) for *e* ∈ *E*. This shows *F*_*A*_ *qF*_*B*_.

(5) It is obvious from (1).

(6) Let *e*_*α*_^*f*^, *F*_*A*_ ∈ *FPS*(*X*, *E*) and *e*_*α*_^*f*^ *qF*_*A*_. Then *α* + *μ*_*A*_(*e*) > 1 or *f*(*e*) is not subset of *f*_*A*_^*c*^(*e*). Since $F_{A}\tilde{\subseteq }F_{B}$, *α* + *μ*_*B*_(*e*) > 1 or *f*(*e*) is not subset of *f*_*B*_^*c*^(*e*). Hence we have *e*_*α*_^*f*^ *qF*_*B*_.

**Proposition 16**

Let {*F*_*A*_*i*__ : *i* ∈ *J*} be a family of FP-soft sets in *FPS*(*X*, *E*), where *J* is an index set. Then *e*_*α*_^*f*^ is FP-soft quasi-coincident with ${\tilde{\cup }}_{i\in J}F_{A_{i}}$ if and only if there exists some *F*_*A*_*i*__ ∈ {*F*_*A*_*i*__ : *i* ∈ *J*} such that *e*_*α*_^*f*^ *qF*_*A*_*i*__.

**Proof**

Obvious.

**Definition 17**

Let FPS(*X*, *E*) and FPS(*Y*, *K*) be families of all FP-soft sets over *X* and *Y*, respectively. Let *u* : *X* → *Y* and *p* : *E* → *K* be two functions. Then a FP-soft mapping *f*_*up*_ : FPS(*X*, *E*) → FPS(*Y*, *K*) is defined as follows.

(1) For *F*_*A*_ ∈ FPS(*X*, *E*), the image of *F*_*A*_ under the FP-soft mapping *f*_*up*_ is the FP-soft set *G*_*S*_ over *Y* defined by the approximate function, ∀*k* ∈ *K*,
  (5) $$\begin{array}{c} g_{S}\left(k\right)=\{\begin{array}{cc} \underset{e\in p^{-1}\left(k\right)}{\cup }u\left(f_{A}\left(e\right)\right), & \text{if}\;\;p^{-1}\left(k\right)\neq ⌀;\\ ⌀, & \text{otherwise}, \end{array} \end{array}$$

where *p*(*A*) = *S* is fuzzy set in *K*.

(2) For *G*_*S*_ ∈ FPS(*Y*, *K*), then the preimage of *G*_*S*_ under the FP-soft mapping *f*_*up*_ is the FP-soft set *F*_*A*_ over *X* defined by the approximate function,  ∀*e* ∈ *E*,
  (6) $$\begin{array}{c} f_{A}\left(e\right)=u^{-1}\left(g_{S}\left(p\left(e\right)\right)\right), \end{array}$$

where *p* ^−1^(*S*) = *A* is fuzzy set in *E*.

If *u* and *p* are injective, then the FP-soft mapping *f*_*up*_ is said to be injective. If *u* and *p* are surjective, then the FP-soft mapping *f*_*up*_ is said to be surjective. The FP-soft mapping *f*_*up*_ is called constant, if *u* and *p* are constant.

**Theorem 18**

Let *X* and *Y* be crips sets *F*_*A*_, *F*_*A*_*i*__ ∈ *FPS*(*X*, *E*), *G*_*S*_, *G*_*S*_*i*__ ∈ *FPS*(*Y*, *K*)  ∀*i* ∈ *J*, where *J* is an index set. Let *f*_*up*_ : *FPS*(*X*, *E*) → *FPS*(*Y*, *K*) be a FP-soft mapping. Then,

1. if  $F_{A_{1}}\tilde{\subset }F_{A_{2}}$ then $f_{up}(F_{A_{1}})\tilde{\subset }f_{up}(F_{A_{2}})$;
2. if $\;G_{S_{1}}\tilde{\subset }G_{S_{2}}$ then $f_{up}^{-1}(G_{S_{1}})\tilde{\subset }f_{up}^{-1}(G_{S_{2}})$;
3. $F_{A}\tilde{\subset }f_{up}^{-1}(f_{up}(F_{A}))$; the equality holds if *f*_*up*_ is injective;
4. $f_{up}(f_{up}^{-1}(G_{S}))\tilde{\subset }G_{S}$; the equality holds if *f*_*up*_ is surjective;
5. $f_{up}({\tilde{\cup }}_{i\in J}F_{A_{i}})={\tilde{\cup }}_{i\in J}f_{up}(F_{A_{i}})$;
6. $f_{up}({\tilde{\cap }}_{i\in J}F_{A_{i}})\tilde{\subset }{\tilde{\cap }}_{i\in J}f_{up}(F_{A_{i}})$; the equality holds if *f*_*up*_ is injective;
7. $f_{up}^{-1}({\tilde{\cup }}_{i\in J}G_{S_{i}})={\tilde{\cup }}_{i\in J}f_{up}^{-1}(G_{S_{i}})$;
8. $f_{up}^{-1}({\tilde{\cap }}_{i\in J}G_{S_{i}})={\tilde{\cap }}_{i\in J}f_{up}^{-1}(G_{S_{i}})$;
9. (*f*_*up*_^−1^(*G*_*S*_))^*c*^ = *f*_*up*_^−1^(*G*_*S*_^*c*^);
10. ${\left(f_{up}(F_{A})\right)}^{c}\tilde{\subset }f_{up}(F_{A}^{c})$;
11. $f_{up}^{-1}(G_{\tilde{K}})=F_{\tilde{E}}$;
12. *f*_*up*_^−1^(*G*_*⌀*_) = *F*_*⌀*_;
13. $f_{up}(F_{\tilde{E}})\tilde{\subset }G_{\tilde{K}}$; the equality holds if *f*_*up*_ is surjective;
14. *f*_*up*_(*F*_*⌀*_) = *G*_*⌀*_.

**Proof**

We only prove (3), (5), (7), (9), (11), and (12). The others can be proved similarly.

(3) Put *G*_*S*_ = *f*_*up*_(*F*_*A*_) and *F*_*B*_ = *f*_*up*_^−1^(*G*_*S*_). Since *A* ≤ *p* ^−1^(*p*(*A*)) = *p* ^−1^(*S*) = *B*, it is sufficient to show that *f*_*A*_(*e*)⊆*f*_*B*_(*e*) for all *e* ∈ *E*,

(7) fB(e)=u−1(gS(p(e)))=u−1(∪e∈p−1(p(e))u(fA(e)))=∪e∈p−1(p(e))u−1(u(fA(e)))⊇fA(e).

This completes the proof.

(5) Put *G*_*S*_*i*__ = *f*_*up*_(*F*_*A*_*i*__) and $G_{S}=f_{up}({\tilde{\cup }}_{i\in J}(F_{A_{i}}))$. Then *S* = *p*(∨*A*_*i*_) = ∨*p*(*A*_*i*_) = ∨*S*_*i*_ and for all *k* ∈ *K*,

(8) gS(k)={∪e∈p−1(k)u(∪i∈JfAi(e))if  p−1(k)≠⌀;⌀otherwise={∪e∈p−1(k)  ∪i∈Ju(fAi(e))if  p−1(k)≠⌀;⌀otherwise={∪i∈J  ∪e∈p−1(k)u(fAi(e))if  p−1(k)≠⌀;⌀otherwise=∪i∈J{∪e∈p−1(k)u(fAi(e))if  p−1(k)≠⌀;⌀otherwise=∪i∈JgSi(k).

This completes the proof.

(7) Put *F*_*A*_*i*__ = *f*_*up*_^−1^(*G*_*S*_*i*__) and $F_{A}=f_{up}^{-1}({\tilde{\cup }}_{i\in J}G_{S_{i}})$. Then *A* = *p* ^−1^(∨*S*_*i*_) = ∨*p* ^−1^(*S*_*i*_) = ∨*A*_*i*_ and for all *e* ∈ *E*,

(9) fA(e)=u−1(∪i∈IgSi(p(e)))=∪i∈Iu−1(gSi(p(e)))=∪i∈JfAi(e).

This completes the proof.

(9) Put *f*_*up*_^−1^(*G*_*S*_) = *F*_*A*_ and *f*_*up*_^−1^(*G*_*S*_^*c*^) = *F*_*B*_. Then for all *e* ∈ *E*,

(10) $$\begin{array}{c} f_{B}\left(e\right)=f_{p^{-1}(S^{c})}\left(e\right)=f_{{\left(p^{-1}(S)\right)}^{c}}\left(e\right)=f_{A^{c}}\left(e\right), \end{array}$$

where *p* ^−1^(*S*) and *p* ^−1^(*S* ^*c*^) are fuzzy sets over *E*. This shows that the approximate functions of *F*_*B*_ and *F*_*A*_^*c*^ are equal. This completes the proof.

(11) Put $F_{A}=f_{up}^{-1}(G_{\tilde{K}})$. Then for all *e* ∈ *E*,

(11) $$\begin{array}{c} f_{A}\left(e\right)=u^{-1}\left(G_{\tilde{K}}\left(p\left(e\right)\right)\right)=u^{-1}\left(Y\right)=X=f_{E}\left(e\right). \end{array}$$

This shows that $F_{A}=F_{\tilde{E}}$.

(12) Since *p* ^−1^(*K*) is fuzzy empty set, that is, 0_*E*_, the proof is clear.

## 4. FP-Soft Topological Spaces

**Definition 19**

A FP-soft topological space is a pair (*X*, *τ*), where *X* is a nonempty set and *τ* is a family of FP-soft sets over *X* satisfying the following properties:

(T1) $F_{⌀},F_{\tilde{E}}\in \tau$;
(T2) if *F*_*A*_, *F*_*B*_ ∈ *τ*, then $F_{A}\tilde{\cap }F_{B}\in \tau$;
(T3) if *F*_*A*_*i*__ ∈ *τ*, ∀*i* ∈ *J*, then ${\tilde{\cup }}_{i\in J}F_{A_{i}}\in \tau$.

*τ* is called a topology of FP-soft sets on *X*. Every member of *τ* is called FP-soft open in (*X*, *τ*). *F*_*B*_ is called FP-soft closed in (*X*, *τ*) if *F*_*B*_^*c*^ ∈ *τ*.

**Example 20**

$\tau_{\text{indiscrete}}=\{F_{⌀},F_{\tilde{E}}\}$ is a FP-soft topology on *X*.

*τ*_discrete_ = FPS(*X*, *E*) is a FP-soft topology on *X*.

**Example 21**

Assume that *X* = {*x*_1_, *x*_2_, *x*_3_, *x*_4_} is a universal set and *E* = {*e*_1_, *e*_2_, *e*_3_} is a set of parameters. If

(12) FA1={((e1)0,2,{x1,x3}),((e2)0,3,{x1,x4}),((e3)0,4,{x2})},FA2={((e1)0,2,{x1,x2,x3}),((e2)0,5,{x1,x4}),((e3)0,4,{x1,x2})},FA3={((e1)0,7,{x1,x3}),((e2)0,3,X),((e3)0,9,{x2,x3})},FA4={((e1)0,7,{x1,x2,x3}),((e2)0,5,X),((e3)0,9,{x2,x3})},

then $\tau =\{F_{⌀},F_{A_{1}},F_{A_{2}},F_{A_{3}},F_{A_{4}},F_{\tilde{E}}\}$ is a FP-soft topology on *X*.

**Theorem 22**

Let (*X*, *τ*) be a FP-soft topological space and let *τ*′ denote family of all closed sets. Then,

1. $F_{⌀},F_{\tilde{E}}\in \tau^{\prime }$;
2. if *F*_*A*_, *F*_*B*_ ∈ *τ*′, then $F_{A}\tilde{\cup }F_{B}\in \tau^{\prime }$;
3. if *F*_*A*_*i*__ ∈ *τ*′, ∀*i* ∈ *J*, then ${\tilde{\cap }}_{i\in J}F_{A_{i}}\in \tau^{\prime }$.

**Proof**

Straightforward.

**Definition 23**

Let (*X*, *τ*) be a FP-soft topological space and *F*_*A*_ ∈ FPS(*X*, *E*). The FP-soft closure of *F*_*A*_ in (*X*, *τ*), denoted by $\bar{F_{A}}$, is the intersection of all FP-soft closed supersets of *F*_*A*_.

Clearly, $\bar{F_{A}}$ is the smallest FP-soft closed set over *X* which contains *F*_*A*_, and $\bar{F_{A}}$ is closed.

**Theorem 24**

Let (*X*, *τ*) be a FP-soft topological space and *F*_*A*_, *F*_*B*_ ∈ *FPS*(*X*, *E*). Then,

1. $\bar{F_{⌀}}=F_{⌀}$ and $\bar{F_{\tilde{E}}}=F_{\tilde{E}}$;
2. $F_{A}\tilde{\subset }\bar{F_{A}}$;
3. $\bar{\bar{F_{A}}}=\bar{F_{A}}$;
4. if $F_{A}\tilde{\subset }F_{B}$, then $\bar{F_{A}}\tilde{\subset }\bar{F_{B}}$;
5. *F*_*A*_ is a FP-soft closed set if and only if $F_{A}=\bar{F_{A}}$;
6. $\bar{F_{A}\tilde{\cup }F_{B}}=\bar{F_{A}}\tilde{\cup }\bar{F_{B}}$.

**Proof**

(1), (2), (3), and (4) are obvious from the definition of FP-soft closure.

(5) Let *F*_*A*_ be a FP-soft closed set. Since $\bar{F_{A}}$ is the smallest FP-soft closed set which contains *F*_*A*_, then $\bar{F_{A}}\tilde{\subset }F_{A}$. Therefore, $F_{A}=\bar{F_{A}}$.

(6) Since $F_{A}\tilde{\subset }F_{A}\tilde{\cup }F_{B}$ and $F_{B}\tilde{\subset }F_{A}\tilde{\cup }F_{B}$, then, by (4), $\bar{F_{A}}\tilde{\subset }\bar{F_{A}\tilde{\cup }F_{B}}$, $\bar{F_{B}}\tilde{\subset }\bar{F_{A}\tilde{\cup }F_{B}}$, and hence $\bar{F_{A}}\tilde{\cup }\bar{F_{B}}\tilde{\subset }\bar{F_{A}\tilde{\cup }F_{B}}$.

Conversely, since $\bar{F_{A}}$ and $\bar{F_{B}}$ are FP-soft closed sets, $\bar{F_{A}}\tilde{\cup }\bar{F_{B}}$ is a FP-soft closed set. Again since $F_{A}\tilde{\cup }F_{B}\tilde{\subset }\bar{F_{A}}\tilde{\cup }\bar{F_{B}}$, by (4), then $\bar{F_{A}\tilde{\cup }F_{B}}\tilde{\subset }\bar{F_{A}}\tilde{\cup }\bar{F_{B}}$.

**Definition 25**

Let (*X*, *τ*) be a FP-soft topological space. A FP-soft set *F*_*A*_ in FPS(*X*, *E*) is called FP-Q-neighborhood (briefly, FP-Q-nbd) of a FP-soft set *F*_*B*_ if there exists a FP-soft open set *F*_*C*_ in *τ* such that *F*_*B*_ *qF*_*C*_ and $F_{C}\tilde{\subseteq }F_{A}$.

**Theorem 26**

Let *e*_*α*_^*f*^, *F*_*A*_ ∈ *FPS*(*X*, *E*). Then $e_{\alpha }^{f}\tilde{\in }\bar{F_{A}}$ if and only if each FP-Q-nbd of *e*_*α*_^*f*^ is FP-soft quasi-coincident with *F*_*A*_.

**Proof**

Let $e_{\alpha }^{f}\tilde{\in }\bar{F_{A}}$. Suppose that *F*_*C*_ is a FP-Q-nbd of *e*_*α*_^*f*^ and $F_{C}\bar{q}F_{A}$. Then there exists a FP-soft open set *F*_*B*_ such that $e_{\alpha }^{f}qF_{B}\tilde{\subseteq }F_{C}$. Since $F_{C}\bar{q}F_{A}$, by Proposition 15(1), $F_{A}\tilde{\subseteq }F_{C}^{c}\tilde{\subseteq }F_{B}^{c}$. Again since *e*_*α*_^*f*^ *qF*_*B*_, *e*_*α*_^*f*^ does not belong to *F*_*B*_^*c*^. This is a contradiction with $\bar{F_{A}}\tilde{\subseteq }F_{B}^{c}$.

Conversely, let each Q-nbd of *e*_*α*_^*f*^ be FP-soft quasi-coincident with *F*_*A*_. Suppose that *e*_*α*_^*f*^ does not belong to $\bar{F_{A}}$. Then there exists a FP-soft closed set *F*_*B*_ which is containing *F*_*A*_ such that *e*_*α*_^*f*^ does not belong to *F*_*B*_. By Proposition 15(5), we have *e*_*α*_^*f*^ *qF*_*B*_^*c*^. Then *F*_*B*_^*c*^ is a FP-Q-nbd of *e*_*α*_^*f*^ and, by Proposition 15(1), $F_{A}\bar{q}F_{B}^{c}$. This is a contradiction with the hypothesis.

**Definition 27**

Let (*X*, *τ*) be a FP-soft topological space and *F*_*A*_ ∈ FPS(*X*, *E*). The FP-soft interior of *F*_*A*_ denoted by *F*_*A*_° is the union of all FP-soft open subsets of *F*_*A*_.

Clearly, *F*_*A*_° is the largest fuzzy soft open set contained in *F*_*A*_, and *F*_*A*_° is FP-soft open.

**Theorem 28**

Let (*X*, *τ*) be a FP-soft topological space and *F*_*A*_, *F*_*B*_ ∈ *FPS*(*X*, *E*). Then,

1. (*F*_*⌀*_)° = *F*_*⌀*_ and ${\left(F_{\tilde{E}}\right)}^{{}^{\circ}}=F_{\tilde{E}}$;
2. $F_{A}^{{}^{\circ}}\tilde{\subset }F_{A}$;
3. (*F*_*A*_°)° = *F*_*A*_°;
4. if $F_{A}\tilde{\subset }F_{B}$, then $F_{A}^{{}^{\circ}}\tilde{\subset }F_{B}^{{}^{\circ}}$;
5. *F*_*A*_ is a FP-soft open set if and only if *F*_*A*_ = *F*_*A*_°;
6. ${\left(F_{A}\tilde{\cap }F_{B}\right)}^{{}^{\circ}}=F_{A}^{{}^{\circ}}\tilde{\cap }F_{B}^{{}^{\circ}}$.

**Proof**

Similar to that of Theorem 24.

**Theorem 29**

Let (*X*, *τ*) be a FP-soft topological space and *F*_*A*_ ∈ *FPS*(*X*, *E*). Then,

1. ${\left(F_{A}^{{}^{\circ}}\right)}^{c}=\bar{F_{A}^{c}}$;
2. ${\left(\bar{F_{A}}\right)}^{c}={\left(F_{A}^{c}\right)}^{{}^{\circ}}$.

**Proof**

We only prove (1). The other is similar. Consider

(13) (FA°)c=(∪~{FB ∣ FB∈τ,FA⊂~FB})c=∩~{FBc ∣ FB∈τ,FA⊂~FB}=∩~{FBc ∣ FBc∈τ′,FBc⊂~FAc}=FAc¯.

**Theorem 30**

Let *c* : *FPS*(*X*, *E*) → *FPS*(*X*, *E*) be an operator satisfying the following:

(c1) *c*(*F*_*⌀*_) = *F*_*⌀*_;
(c2) $F_{A}\tilde{\subseteq }c(F_{A}),\forall F_{A}\in FPS(X,E)$;
(c3) $c(F_{A}\tilde{\cup }F_{B})=c(F_{A})\tilde{\cup }c(F_{B}),\forall F_{A},F_{B}\in FPS(X,E)$;
(c4) *c*(*c*(*F*_*A*_)) = *c*(*F*_*A*_), ∀*F*_*A*_ ∈ *FPS*(*X*, *E*).

Then one can associate FP-soft topology in the following way:

(14) $$\begin{array}{c} \tau =\left\{F_{A}^{c}\in FPS\left(X,E\right)\;|\;c\left(F_{A}\right)=F_{A}\right\}. \end{array}$$

Moreover with this FP-soft topology *τ*, $\bar{F_{A}}=c(F_{A})$ for every *F*_*A*_ ∈ *FPS*(*X*, *E*).

**Proof**

(T1) By (c1), $F_{⌀}^{c}=F_{\tilde{E}}\in \tau$. By (c2) $F_{\tilde{E}}\tilde{\subseteq }c(F_{\tilde{E}})$, so $c(F_{\tilde{E}})=F_{\tilde{E}}$ and *F*_*⌀*_ ∈ *τ*.

(T2) Let *F*_*A*_, *F*_*B*_∈*τ*. By the definition of *τ*, *c*(*F*_*A*_^*c*^) = *F*_*A*_^*c*^ and *c*(*F*_*B*_^*c*^) = *F*_*B*_^*c*^. By (c3), $c({\left(F_{A}\tilde{\cap }F_{B}\right)}^{c})=c(F_{A}^{c}\tilde{\cup }F_{B}^{c})=c(F_{A}^{c})\tilde{\cup }c(F_{B}^{c})=F_{A}^{c}\tilde{\cup }F_{B}^{c}={\left(F_{A}\tilde{\cap }F_{B}\right)}^{c}$. So $F_{A}\tilde{\cap }F_{B}\in \tau$.

(T3) Let {*F*_*A*_*i*__ | *i* ∈ *J*} ⊂ *τ*. Since *c* is order preserving and ${\tilde{\cap }}_{i\in J}^{}F_{A_{i}}^{c}\tilde{\subseteq }F_{A_{k}}^{c}$,∀*k* ∈ *J*, then $c({\tilde{\cap }}_{i\in J}^{}F_{A_{i}}^{c})\tilde{\subseteq }c(F_{A_{k}}^{c})=F_{A_{k}}^{c}$. Then we have $c({\tilde{\cap }}_{i\in J}^{}F_{A_{i}}^{c})\tilde{\subseteq }{\tilde{\cap }}_{i\in J}^{}F_{A_{i}}^{c}$. Conversely, by (c2) we have ${\tilde{\cap }}_{i\in J}^{}F_{A_{i}}^{c}\tilde{\subseteq }c({\tilde{\cap }}_{i\in J}^{}F_{A_{i}}^{c})$. Hence, $c({\left({\tilde{\cup }}_{i\in J}^{}F_{A_{i}}\right)}^{c})=c({\tilde{\cap }}_{i\in J}^{}F_{A_{i}}^{c})={\tilde{\cap }}_{i\in J}^{}F_{A_{i}}^{c}={\left({\tilde{\cup }}_{i\in J}^{}F_{A_{i}}\right)}^{c}$ and ${\tilde{\cup }}_{i\in J}^{}F_{A_{i}}\in \tau$.

Now we will show that with this FP-soft topology *τ*, $\bar{F_{A}}=c(F_{A})$ for every *F*_*A*_ ∈ FPS(*X*, *E*). Let *F*_*A*_ ∈ FPS(*X*, *E*). Since ${\left(\bar{F_{A}}\right)}^{c}\in \tau$, then $c(\bar{F_{A}})=\bar{F_{A}}$. Since *c* is order preserving, $c(F_{A})\tilde{\subseteq }c(\bar{F_{A}})=\bar{F_{A}}$. Conversely, by (c4) we have (*c*(*F*_*A*_))^*c*^ ∈ *τ*. Then since $F_{A}\tilde{\subseteq }c(F_{A})$ and $\bar{F_{A}}$ is the smallest FP-soft closed set over *X* which contains *F*_*A*_, $\bar{F_{A}}\tilde{\subseteq }c(F_{A})$.

The operator *c* is called the FP-soft closure operator.

**Remark 31**

By Theorem 24(1), (2), (3), and (6) and Theorem 30, we see that with a FP-soft closure operator we can associate a FP-soft topology and conversely with a given FP-soft topology we can associate a FP-soft closure operator.

**Theorem 32**

Let *i* : *FPS*(*X*, *E*) → *FPS*(*X*, *E*) be an operator satisfying the following:

(i1) $i(F_{\tilde{E}})=F_{\tilde{E}}$;
(i2) $i(F_{A})\tilde{\subseteq }F_{A}$, ∀*F*_*A*_ ∈ *FPS*(*X*, *E*);
(i3) $i(F_{A}\tilde{\cap }F_{B})=i(F_{A})\tilde{\cap }i(F_{B})$, ∀*F*_*A*_, *F*_*B*_ ∈ *FPS*(*X*, *E*);
(i4) *i*(*i*(*F*_*A*_)) = *i*(*F*_*A*_), ∀*F*_*A*_ ∈ *FPS*(*X*, *E*).

Then one can associate a FP-soft topology in the following way:

(15) $$\begin{array}{c} \tau =\left\{F_{A}\in FPS\left(X,E\right)\;|\;i\left(F_{A}\right)=F_{A}\right\}. \end{array}$$

Moreover, with this fuzzy soft topology *τ*, (*F*_*A*_)° = *i*(*F*_*A*_) for every *F*_*A*_ ∈ *FPS*(*X*, *E*).

**Proof**

Similar to that of Theorem 30.

The operator *i* is called the FP-soft interior operator.

**Remark 33**

By Theorem 28(1), (2), (3), and (6) and Theorem 32, we see that with a FP-soft interior operator we can associate a FP-soft topology and conversely with a given FP-soft topology we can associate a FP-soft interior operator.

**Definition 34**

Let (*X*, *τ*) be a FP-soft topological space. A subcollection *B* of *τ* is called a base for *τ* if every member of *τ* can be expressed as a union of members of *B*.

**Example 35**

If we consider the FP-soft topology *τ* in Example 21, then one easily sees that the family $B=\{F_{⌀},F_{A_{1}},F_{A_{2}},F_{A_{3}},F_{\tilde{E}}\}$ is a basis for *τ*.

**Proposition 36**

Let (*X*, *τ*) be a FP-soft topological space and *B* is subfamily of *τ*. *B* is a base for *τ* if and only if for each *e*_*α*_^*f*^ in *FPS*(*X*, *E*) and for each FP-soft open Q-nbd *F*_*A*_ of *e*_*α*_^*f*^, there exists a *F*_*B*_ ∈ *B* such that $e_{\alpha }^{f}qF_{B}\tilde{\subseteq }F_{A}$.

**Proof**

Let *B* be a base for *τ*, $e_{\alpha }^{f}\tilde{\in }\;\text{FPS}(X,E)$ and let *F*_*A*_ be a FP-soft open Q-nbd of *e*_*α*_^*f*^. Then there exists a subfamily *B*′ of *B* such that $F_{A}=\tilde{\cup }\{F_{B}|F_{B}\in B^{\prime }\}$. Suppose that $e_{\alpha }^{f}\bar{q}F_{B}$ for all *F*_*B*_ ∈ *B*′. Then *α* + *μ*_*B*_(*e*) ≤ 1 and *f*(*e*)⊆*f*_*B*_^*c*^(*e*) for every *F*_*B*_ ∈ *B*′. Therefore, we have *α* + *μ*_*A*_(*e*) ≤ 1 and *f*(*e*) is not subset of *f*_*A*_(*e*) since *μ*_*A*_(*e*) = sup⁡{*μ*_*B*_(*e*) | *F*_*B*_ ∈ *B*′} and *f*_*A*_(*e*) = ∪*f*_*B*_(*e*). This is a contradiction.

Conversely, if *B* is not a base for *τ*, then there exists a *F*_*A*_ ∈ *τ* such that $F_{C}=\tilde{\cup }\{F_{B}\in B:F_{B}\tilde{\subseteq }F_{A}\}\neq F_{A}$. Since *F*_*C*_ ≠ *F*_*A*_, there exists *e* ∈ *E* such that *μ*_*C*_(*e*) < *μ*_*A*_(*e*) or *f*_*C*_(*e*) ⊂ *f*_*A*_(*e*). Put *α* = 1 − *μ*_*C*_(*e*) and *f*(*e*) = *f*_*C*_^*c*^(*e*). Then in both cases, we obtain *e*_*α*_^*f*^ *qF*_*A*_ and $e_{\alpha }^{f}\bar{q}F_{C}$. Therefore, we have *μ*_*B*_(*e*) + *α* ≤ *μ*_*C*_(*e*) + *α* = 1 and *f*(*e*)∩*f*_*B*_(*e*)⊆*f*(*e*)∩*f*_*C*_(*e*) = *⌀*, that is, $e_{\alpha }^{f}\bar{q}F_{B}$ for all *F*_*B*_ ∈ *B* which is contained in *F*_*A*_. This is a contradiction.

**Definition 37**

Let (*X*, *τ*_1_) and (*Y*, *τ*_2_) be two FP-soft topological spaces. A FP-soft mapping *f*_*up*_ : (*X*, *τ*_1_)→(*Y*, *τ*_2_) is called FP-soft continuous if *f*_*up*_^−1^(*G*_*S*_) ∈ *τ*_1_, ∀*G*_*S*_ ∈ *τ*_2_.

**Example 38**

Assume that *X* = {*x*_1_, *x*_2_, *x*_3_}, *Y* = {*y*_1_, *y*_2_, *y*_3_} are two universal sets, *E* = {*e*_1_, *e*_2_}, *K* = {*k*_1_, *k*_2_} are two parameter sets, and *f*_*up*_ : (*X*, *τ*_1_)→(*Y*, *τ*_2_) is a FP-soft mapping, where *u*(*x*_1_) = *y*_2_, *u*(*x*_2_) = *y*_1_, *u*(*x*_3_) = *y*_3_, and *p*(*e*_1_) = *k*_2_ and *p*(*e*_2_) = *k*_1_. If we take *F*_*A*_ = {((*e*_1_)_0,3_, {*x*_2_, *x*_3_}), ((*e*_2_)_0,2_, {*x*_1_, *x*_2_})}, *G*_*S*_ = {((*k*_1_)_0,2_, {*y*_1_, *y*_2_}), ((*k*_2_)_0,3_, {*y*_1_, *y*_3_})}, *τ*_1_ = $\left\{F_{⌀},F_{\tilde{E}},F_{A}\right\}$, and *τ*_2_ = $\left\{{\tilde{0}}_{K},{\tilde{1}}_{K},G_{S}\right\}$, then *f*_*up*_ is a FP-soft continuous mapping.

The constant mapping *f*_*up*_ : (*X*, *τ*_1_)→(*Y*, *τ*_2_) is not continuous in general.

**Example 39**

Assume that *X* = {*x*_1_, *x*_2_, *x*_3_}, *Y* = {*y*_1_, *y*_2_} are two universal sets, *E* = {*e*_1_, *e*_2_}, *K* = {*k*_1_, *k*_2_} are two parameter sets, and *f*_*up*_ : (*X*, *τ*_1_)→(*Y*, *τ*_2_) is a constant FP-soft mapping, where *u*(*x*_1_) = *u*(*x*_2_) = *u*(*x*_3_) = *y*_2_ and *p*(*e*_1_) = *p*(*e*_2_) = *k*_1_. If we take *G*_*S*_ = {((*k*_1_)_0,2_, {*y*_1_, *y*_2_}), ((*k*_2_)_0,5_, {*y*_2_, *y*_3_})}, *τ*_1_ = $\left\{F_{⌀},F_{\tilde{E}}\right\}$, and *τ*_2_ = $\left\{F_{⌀},F_{\tilde{K}},G_{S}\right\}$, *f*_*up*_ is not a FP-soft continuous since *f*_*up*_^−1^(*G*_*S*_) ∉ *τ*_1_.

Let *α* ∈ [0,1]. We denote by *α*_*E*_ the constant fuzzy set on *E*; that is, *μ*_*α*_*E*__(*e*) = *α* for all *e* ∈ *E* and *α* ∈ [0,1].

**Definition 40**

Let *F*_*A*_ ∈ FPS(*X*, *E*). *F*_*A*_ is called *α*-*A*-universal FP-soft set if *μ*_*A*_(*e*) = *α* and *f*_*A*_(*e*) = *X* for all *e* ∈ *A*, denoted by $F_{{\tilde{\alpha }}_{A}}$.

**Definition 41 (see [21])**

A FP-soft topology is called enriched if it satisfies $F_{{\tilde{\alpha }}_{A}}\in \tau$ and $F_{{\tilde{\alpha }}_{A}}^{c}\in \tau$ for all *α* ∈ (0,1].

**Theorem 42**

Let (*X*, *τ*_1_) be an enriched FP-soft topological space, (*Y*, *τ*_2_) a FP-soft topological space, and *f*_*up*_ : *FPS*(*X*, *E*) → *FPS*(*Y*, *K*) a constant FP-soft mapping. Then *f*_*up*_ is FP-soft continuous.

**Proof**

Let *G*_*S*_ ∈ *τ*_2_. Put *f*_*up*_^−1^(*G*_*S*_) = *F*_*A*_. Then *A* = *p* ^−1^(*S*) = *α*_*E*_, where *α* = sup⁡_*k*∈*K*_{*μ*_*S*_(*k*)} and

(16) $$\begin{array}{c} f_{A}\left(e\right)=u^{-1}\left(g_{S}\left(p\left(e\right)\right)\right)=\{\begin{array}{cc} X, & g_{S}\left(p\left(e\right)\right)\neq ⌀\\ ⌀, & \text{otherwise} \end{array} \end{array}$$

for all *e* ∈ *E*. Hence $F_{A}=F_{{\tilde{\alpha }}_{E}}\in \tau_{1}$ or $F_{A}=F_{{\tilde{\alpha }}_{E}}^{c}\in \tau_{1}$ and so *f*_*up*_ : (*X*, *τ*_1_)→(*Y*, *τ*_2_) is FP-soft continuous.

**Theorem 43**

Let (*X*, *τ*_1_) and (*Y*, *τ*_2_) be two FP-soft topological spaces and let *f*_*up*_ : *FPS*(*X*, *E*) → *FPS*(*Y*, *K*) be a FP-soft mapping. Then the following are equivalent:    

1. *f*_*up*_ is FP-soft continuous;
2. *f*_*up*_^−1^(*G*_*S*_) is FP-soft closed for every FP-closed set *G*_*S*_ over *Y*;
3. $f_{up}(\bar{F_{A}})\tilde{\subset }\bar{f_{up}(F_{A})}$, ∀*F*_*A*_ ∈ *FPS*(*X*, *E*);
4. $\bar{f_{up}^{-1}(G_{S})}\tilde{\subset }f_{up}^{-1}(\bar{G_{S}})$, ∀*G*_*S*_ ∈ *FPS*(*Y*, *K*);
5. $f_{up}^{-1}{\left(G_{S}^{{}^{\circ}})\tilde{\subset }(f_{up}^{-1}(G_{S})\right)}^{{}^{\circ}}$, ∀*G*_*S*_ ∈ *FPS*(*Y*, *K*).

**Proof**

(1)⇒(2) It is obvious from Theorem 18(9).

(2)⇒(3) Let *F*_*A*_ ∈ FPS(*X*, *E*). Since $F_{A}\tilde{\subset }f_{up}^{-1}(f_{up}(F_{A}))F_{A}\tilde{\subset }f_{up}^{-1}(\bar{f_{up}(F_{A})})\in \tau_{1}^{\prime }$, therefore we have $\bar{F_{A}}\tilde{\subset }f_{up}^{-1}(\bar{f_{up}(F_{A})})$. By Theorem 18(4), we get $f_{up}(\bar{F_{A}})\tilde{\subset }$ $f_{up}(f_{up}^{-1}(\bar{f_{up}(F_{A})})\tilde{\subset }\bar{f_{up}(F_{A})}$.

(3)⇒(4) Let *G*_*S*_ ∈ FPS(*Y*, *K*). If we choose *f*_*up*_^−1^(*G*_*S*_) instead of *F*_*A*_ in (3), then $f_{up}(\bar{f_{up}^{-1}(G_{S})})\tilde{\subset }\bar{f_{up}(f_{up}^{-1}(G_{S}))}\tilde{\subset }\bar{G_{S}}$. Hence by Theorem 18(3), $\bar{f_{up}^{-1}(G_{S})}\tilde{\subset }f_{up}^{-1}(f_{up}(\bar{f_{up}^{-1}(G_{S})}))$ $\tilde{\subset }f_{up}^{-1}(\bar{G_{S}})$.

(4)⇔(5) These follow from Theorem 18(9) and Theorem 29.

(5)⇒(1) Let *G*_*S*_ ∈ *τ*_2_. Since *G*_*S*_ is a FP-soft open set, then $f_{up}^{-1}(G_{S})=f_{up}^{-1}{\left(G_{S}^{{}^{\circ}})\tilde{\subset }(f_{up}^{-1}(G_{S})\right)}^{{}^{\circ}}\tilde{\subset }f_{up}^{-1}(G_{S})$. Consequently, *f*_*up*_^−1^(*G*_*S*_) is a FP-soft open and so *f*_*up*_ is FP-soft continuous.

**Theorem 44**

Let *f*_*up*_:(*X*, *τ*_1_)→(*Y*, *τ*_2_) be a FP-soft mapping and let *B* be a base for *τ*_2_. Then *f*_*up*_ is FP-soft continuous if and only if *f*_*up*_^−1^(*G*_*S*_) ∈ *τ*_1_, ∀*G*_*S*_ ∈ *B*.

**Proof**

Straightforward.

**Definition 45**

A family *C* of FP-soft sets is a cover of a FP-soft set *F*_*A*_ if $F_{A}\tilde{\subseteq }\tilde{\cup }\{F_{A_{i}}:F_{A_{i}}\in C,i\in J\}$. It is a FP-soft open cover if each member of *C* is a FP-soft open set. A subcover of *C* is a subfamily of *C* which is also a cover.

**Definition 46**

A family *C* of FP-soft sets has the finite intersection property if the intersection of the members of each finite subfamily of *C* is not empty FP-soft set.

**Definition 47**

A FP-soft topological space (*X*, *τ*) is FP-compact if each FP-soft open cover of $F_{\tilde{E}}$ has a finite subcover.

**Example 48**

Let *X* = {*x*_1_, *x*_2_,…}, *E* = {*e*_1_, *e*_2_,…}, and *F*_*A*_*n*__ = {((*e*_*i*_)_1/*n*_, *X* − {*x*_1_, *x*_2_,…, *x*_*n*_}) : *i* = 1,2,…}. Then $\tau =\{F_{A_{n}}:n=1,2,\ldots \}\cup \{F_{⌀},\;F_{\tilde{E}}\}$ is a FP-soft topology on *X*, and (*X*, *τ*) is FP-compact.

**Theorem 49**

A FP-soft topological space is FP-soft compact if and only if each family of FP-soft closed sets with the finite intersection property has a nonempty FP-soft intersection.

**Proof**

If *C* is a family of FP-soft sets in a FP-soft topological space (*X*, *τ*), then *C* is a cover of $F_{\tilde{E}}$ if and only if one of the following conditions hold:

1. $\tilde{\cup }\{F_{A_{i}}:F_{A_{i}}\in C,i\in J\}=$ $F_{\tilde{E}}$;
2. ${\left(\tilde{\cup }\{F_{A_{i}}:F_{A_{i}}\in C,i\in J\}\right)}^{c}=$ $F_{\tilde{E}}^{c}=F_{⌀}$;
3. $\tilde{\cap }\{F_{A_{i}}^{c}:F_{A_{i}}\in C,i\in J\}=F_{⌀}$.

Hence the FP-soft topological space is FP-soft compact if and only if each family of FP-soft open sets over *X* such that no finite subfamily covers $F_{\tilde{E}}$ fails to be a cover, and this is true if and only if each family of FP-soft closed sets which has the finite intersection property has a nonempty FP-soft intersection.

**Theorem 50**

Let (*X*, *τ*_1_) and (*Y*, *τ*_2_) be FP-soft topological spaces and let *f*_*up*_ : *FPS*(*X*, *E*) → *FPS*(*Y*, *K*) be a FP-soft mapping. If (*X*, *τ*_1_) is FP-soft compact and *f*_*up*_ is FP-soft continuous surjection, then (*Y*, *τ*_2_) is FP-soft compact.

**Proof**

Let *C* = {*G*_*S*_*i*__ : *i* ∈ *J*} be a cover of $G_{\tilde{K}}$ by FP-soft open sets. Then since *f*_*up*_ is FP-soft continuous, the family of all FP-soft sets of the form *f*_*up*_^−1^(*G*_*S*_), for *G*_*S*_ ∈ *C*, is a FP-soft open cover of $F_{\tilde{E}}$ which has a finite subcover. However, since *f*_*up*_ is surjective, then *f*_*up*_(*f*_*up*_^−1^(*G*_*S*_)) = *G*_*S*_ for any FP-soft set *G*_*S*_ over *Y*. Thus, the family of images of members of the subcover is a finite subfamily of *C* which covers $G_{\tilde{K}}$. Consequently, (*Y*, *τ*_2_) is FP-soft compact.

## 5. Conclusion

Topology is a branch of mathematics, whose concepts exist not only in almost all branches of mathematics, but also in many real life applications. In this paper, we introduce the topological structure of fuzzy parametrized soft sets and fuzzy parametrized soft mappings. We study some fundamental concepts in fuzzy parametrized soft topological spaces such as closures, interiors, bases, compactness, and continuity. Some basic properties of these concepts are also presented. This paper will form the basis for further applications of topology.
